# *Ocimum gratissimum*: Chemical Composition, Phytochemical Properties, Antioxidants, and Pharmacological Activities: A Review

**DOI:** 10.3390/plants15111662

**Published:** 2026-05-28

**Authors:** Nhlanhla Maphetu, Jeremiah O. Unuofin, Adewale O. Oladipo, Sogolo L. Lebelo

**Affiliations:** 1 Department of Life and Consumer Sciences, College of Agriculture and Environmental Sciences, University of South Africa, Private Box X06, Florida 1710, South Africa; bonganimapheto@gmail.com (N.M.); lebelol@unisa.ac.za (S.L.L.); 2 UNISA Biomedical Engineering Research Group, Department of Mechanical, Bioresources and Biomedical Engineering, College of Science, Engineering and Technology (CSET), University of South Africa, Florida 1710, South Africa; walexy20002001@gmail.com

**Keywords:** *Ocimum gratissimum*, African basil, bioactive compounds, pharmacological mechanisms, therapeutic activities

## Abstract

*Ocimum gratissimum* L. subsp., commonly known as African basil, is a native African medicinal plant that has been used for generations to address various health issues. These include colds, flu, diabetes, diarrhoea, pain and swelling, psychological disorders, malaria, inflammation, and infections caused by fungi and bacteria. In addition, African basil is abundant in vitamins and minerals and is mostly used to add flavour to dishes and soups in West African households. Studies have identified multiple bioactive compounds in this plant, such as alkaloids, polyphenols, triterpenes, steroids, fatty acids, esters, alcohols, essential oils, ketones, and aldehydes. Key bioactive constituents, essential oils like thymol and eugenol, are responsible for the pharmacological effects of *Ocimum gratissimum*. The diverse bioactive compounds give the plant a wide range of therapeutic properties, including antioxidant, cognitive-enhancing, antimicrobial, anti-inflammatory, analgesic, anticancer, antihypertensive, hepatoprotective, and organoleptic effects. Notable mechanisms of action include the PI3K/Akt, NRF-2, and NF-κB signalling pathways, free radical scavenging, and modulation of pro-inflammatory cytokines such as IL-1β, IL-6, and TNF-α. This review paper aimed to compile recent studies on the phytochemistry, medicinal uses, therapeutic activities, and molecular mechanisms of action of *Ocimum gratissimum*. Further studies are needed to better understand the effects of *Ocimum gratissimum* at the pathological and molecular levels.

## 1. Introduction

Africa is home to over 45,000 plant species, and over 5000 of these plants are used as medicinal plants [1]. Medicinal plants are key remedies to treating various ailments such as obesity, heart-related diseases, respiratory diseases, diabetes, cold and flu, kidney complications, infections, and diarrhoea, amongst others [2,3]. Over 4000 of the available medicinal plants belong to the Lamiaceae family in African countries [1]. One of these medicinal plants is the sacred plant, *Ocimum gratissimum*. According to [4], *Ocimum gratissimum* is regarded as a sacred plant because it is used during prayers as one of the essences by the Hindu religion.

The plant, *Ocimum gratissimum*, belongs to the family Lamiaceae (previously Labiatae), and genus *Ocimum* of the species *gratissimum*. *Ocimum gratissimum* L. subsp., is a perennial shrub plant that is regarded as native to most African countries, such as Western Africa, Central and Southern Africa [5,6]. According to [7] revealed that *O. gratissimum* is native to the Asian part of the world [7,8]. Some of the Asian countries where *O. gratissimum* is mostly found are Iran, Nepal, India, and Sri Lanka [9,10]. In India, the Hindi and Nepali-speaking population know *O. gratissimum* as *Ram tulasi* [11] and *Vriddhutulsi* and *Nimma tulasi*, respectively [12]. Additionally, the *O. gratissimum* is widely distributed in West, East and Southern African countries such as Nigeria, Ghana, Kenya, Uganda, Madagascar, and South Africa [6,10,13,14,15].

In South Africa, the *O. gratissimum* plant is distributed in different parts of the country, from the tropicals of KwaZulu-Natal to the inland provinces of Limpopo and Mpumalanga. According to the South African National Biodiversity Institute (SANBI), *O. gratissimum* is known to locals as *Umbijazane*, *Umnandi*, and *Uqabukhulu* in the Zulu language [16,17]. The native names referred to are predominantly in IsiZulu, as the plant is widely distributed in Mpumalanga and KwaZulu-Natal, where most people in these regions are of the Zulu nation [18]. In most English-speaking regions, the plant is known as wild basil [16], scent or leaf basil [19,20,21], while in other African countries and the global market, the *O. gratissimum* is known as the African basil [5,7,15].

*O. gratissimum* is known for its rich aromatic properties [22,23], which are derived from its leaf essential oils [24]. *O. gratissimum* is said to be a herbaceous plant [22], which is mostly appreciated for its magnificent beauty, with decorative and vegetative shiny leaves with blooming flowers [25]. The plant is known for its many medicinal uses and edible benefits.

For instance, folklorically, *O. gratissimum* leaves are used for culinary purposes in Nigeria by adding the plant as part of a salad, or by pouring the plant in a pot to create flavour in a cooked soup [19,26,27]. In addition, it is used for the treatment and management of various ailments, such as diabetes [22], cold and fever, cough, and bronchitis [28], stomach aches and stomach discomfort, epilepsy, nostrils, and abdominal pains [26,29]. Moreover, other uses of *O. gratissimum* include using the plant as a mosquito repellent [30] and for treatment of bacteria and ear infections [31]. According to Ugbogu, *O. gratissimum* is used traditionally in pain management treatment, and the infusion is used to relieve cough (antitussive) [32].

*O. gratissimum* has a wide range of therapeutic uses, such as antioxidants, anti-inflammatory, antibacterial, antimicrobial, antipyretic, antifertility, neuroprotective, cognitive enhancing, and anti-cardiovascular, amongst others [2,31,33,34,35,36]. For example, Alvarenga reported that the presence of essential oils in *O. gratissimum* leaves promotes aromatherapeutic properties [23]. Moreover, essential oils of *O. gratissimum* are said to contain a variety of subsequent bioactive compounds such as eugenol, thymol, and citral, which have antifungal and antiflatoxigenic properties [23,37]. Additionally, *O. gratissimum* has high contents of crude and essential oils, organic acids, terpenes and flavonoids, which have been investigated to treat sickle cell diseases [37]. Furthermore, the essential oils of *O. gratissimum* are referred to as *Ocimum* oils, thyme and clove oils. These oils have been reported to possess anti-inflammatory and antimicrobial properties [10,38]. Additionally, in another study, the leaves of the plant have been reported to contain amines, alkones, aromatic compounds and alkyl compounds [13].

Medicinal plants continue to offer numerous economic and health benefits, driving the demand for more sustainable and efficient therapeutic strategies. The development of modern pharmaceutical products depends on compounds originating from medicinal plants. Although review articles on *Ocimum gratissimum* have already discussed conventional medicinal plants and their general use, several often ignore the convergence of the plant characteristics and scientific validation. Unlike previous reviews, this work examines the data-driven properties of bioactive compounds found in *Ocimum gratissimum*, such as eugenol, essential oils, polyphenols, and vitamins. Interestingly, the structure–activity relationship governing pharmacological activities and molecular mechanism studies that modulate their medicinal properties, as well as the current knowledge gap relating to effective utilisation, were identified. In addition, the review provides a critical analysis of previous research and provides an updated overview of the ethnomedicinal uses, toxicity, phytochemistry, and pharmacological activities of the *Ocimum gratissimum* L. plant.

### Methodology

Several online search engines were used to gather journal papers, dissertations, and reports. The computerised database used to gather information for this review paper includes BMC, Wiley, Taylor & Francis online, PubMed, ScienceDirect and Google Scholar. Dissertations and thesis documents were gathered from the National ETD portal of South African theses and dissertations. Keywords used to generate information were: *O. gratissimum*, *O gratissimum*, *Ocimum gratissimum* L., basil leaf, basil flower, ram talsi, African basil, African leaf, and holy basil, medicinal uses of, ethnological use of, pharmacological use, antioxidant properties of, pharmacological properties, phenolic, phytochemical, anticancerous, antidiabetic, antipyretic, antibacterial, antiviral properties, antifungal, antibacterial, toxicity and antifertility.

## 2. Plant Characteristics

The herbaceous shrub, *O. gratissimum*, is made of five structural components, which are the roots, branches, stem, leaves and flowers. These component characteristics are discussed as follows:

### 2.1. Roots

The *O. gratissimum* roots are characterised by a fibrous root, which thickens and deepens towards the ground [34]. They appear lighter in colour, exhibiting a brown colour with a smooth texture. According to Sharma, *O. gratissimum* roots, similarly to the stem and leaves, have a majority of the pharmacological therapeutic activities such as anti-inflammatory, antioxidant, antidiabetic properties [22,39,40], antiviral [2] and neuroprotective properties [20]. Some of the notable bioactive compounds reported from the root extracts include flavonoids, tannins, phenolics [22,39], ethers, ketones, and hydrocarbons [41]. Additionally, the root portion of the plant has been used to combat various ailments, including sedating children [12,34], nose blockages, abdominal pains, and ear infections [34]. Pandey and colleagues also highlight that the roots of the *Ocimum* plant species assist in improving physical and mental strength when ingested [42].

### 2.2. Branches

The *O. gratissimum* branches are characterised by a dense shape, greenish-brown colour and pubescent appearance [38,43]. The shrub *O. gratissimum* contains multiple branches on each plant, which usually grow up to 1 metre long [44,45]. Identified active compounds from the branch extract include saponins, tannins, phlobatannins [46], essential oils, and other minerals such as calcium, potassium and nitrogen [38,47]. The presence of the bioactive compounds allows the branch extracts to be useful in combating ailments such as fever, cold and flu, and bacterial and fungal infections [47,48]. Some researchers have stated therapeutic activities associated with branch extracts to include antibacterial, antifungal, antihypertensive and antioxidant properties [43,46,49,50].

### 2.3. Stem

*O. gratissimum* is characterised by a woody brown stem, which appears square and short [27,51]. The stem is short as it contains a shrubby structure and can grow up to 2–3 m long [27,52]. The perennial shrub stem appears to be pubescent in young growing trees, and the colour appears to be greener compared to other mature shrub trees [27,51,53]. The base of the stem is firm with the epidermis, which looks like peeling off the strip of its base [50].

The stem portion has been reported to be rich in essential oils, which are used to treat bacterial infection, candidiasis, haemorrhoids, inflammation and chest pains [7,54,55,56]. Some of the therapeutic activities investigated from the stem extract include insecticidal properties [56], antioxidants, antibacterial [51], and antiprotozoal [50]. Furthermore, *O. gratissimum* contains high levels of eugenol essential oil [56], limonene, linalool and camphor bioactive compounds [7,57].

### 2.4. Flowers

The African basil is characterised by blooming colourful flowers, which are often used in gardens across African and Asian homes [7,43,58]. *O. gratissimum* flowers are characterised by a cream-white colour and purplish colour [7,59]. Flower extracts have been reported to have a pungent smell [60]. Flowers appear to be attached to the lapping stem and arranged in whorls [7,61]. In some areas of the world, the flowers appear to be greenish-yellow with elongated racemes, positioned in the axis quadrangular, with dense hair [7,50]. The base of the flower is sessile and appears oval with a bract which is measured at about 3–12 mm and 1–7 mm long [50]. Additionally, the pedicels of the flower appear longer than the calyx, with the calyx being ovoid in shape and measured at 3–4 mm [7,57].

Flower extracts have been identified to contain a variety of pharmacological properties. For instance, in a study by [58], the *O. gratissimum* flowers were used as insect repellents. In other studies, antioxidants, antifungal, antibacterial, and ovoidal activities have been investigated [51,52,58,62]. Moreover, the *O. gratissimum* flower extracts have been vastly investigated as they are rich in essential oils. In one study, ref. [62] report findings of various essential oils from the flower including methyl ester, methyl eugenol, caryophyllene, and linoleic acid [62]. Additionally, some studies have found a prevalent bioactive compound, eugenol, which is responsible for many of the flower’s pharmacological and therapeutic activities [6,63,64,65].

### 2.5. Leaves

The *O. gratissimum* leaves are the most extensively researched part of the plant. The *O. gratissimum* leaves are characterised by their elliptic-ovate shape and arrangement of the two opposite growing leaves from the nodes. Additionally, they have a translucent texture, with serrated margins and share saw-like edges. The leaf base appears wedged in its shape and contains crenate margins, with the apex, which is more tapered and pointier. The *O. gratissimum* leaves grow up to 3–9 cm wide and 5–13 cm long, their colour is green, and with maturity it turns lime. The leaves are the source of the pungent camphoraceous smell of the plant.

*O. gratissimum* leaves have been documented to be used traditionally across the globe for different reasons. To indicate a few, the *O. gratissimum* leaf extracts have been used to treat respiratory infections, stomach and laxative concerns, and ear infections [66,67]. Leaves are also used as condiments to make flour and food due to the presence of minerals such as fibres and carbohydrates [8,68]. Other uses include the treatment of bacterial and microbial infections, diarrhoea, pneumonia, headaches, colds, and flu [23,67,68,69]. *O. gratissimum* leaves are a rich source of antioxidants, and have anti-inflammatory [23,70], antibacterial and anticancerous properties [71]. Furthermore, ref. [27] add that the plant has therapeutic properties associated with neuroprotective potential [27].

The variety of therapeutic properties available from the leaves is due to the presence of multiple bioactive compounds. Moreover, *O. gratissimum* leaves are essential oil rich, with some researchers indicating that the plant is the source of eugenol [6,27,62]. Other reported bioactive compounds from the leaves include alkaloids [67], tannins, saponins, flavonoids, steroids [23,62], anthraquinones, glycosides, and thymol [72,73]. Chanthavong et al. add that the *O. gratissimum* leaves contain amines, aromatic compounds, alkyl and alkanes [13]. Other bioactive compounds indicated include the presence of polyphenols [71], N-propylamine, vernomine, and piperamide [67].

## 3. Minerals and Nutrition

*O. gratissimum* is also a consumable, nutritionally rich plant. Various authors have reported diverse nutritional ways to consume and use it as a flavouring agent. For example, quantitative analysis of minerals shows that the presence of various minerals’ secondary metabolite constituents is significantly higher compared to the recommendations of the World Health Organisation (WHO) consumption values per kilogram concentration [74]. The identified minerals include magnesium (1.17 ± 0.537 mg/kg), zinc (0.20 ± 0.06 mg/kg), sodium (0.31 ± 0.049 mg/kg) and potassium (0.26 ± 0.077 mg/kg) [74,75]. Oyet and Chibor (2024) also researched the preference of spices among various participants, and the findings suggested that individuals preferred medicinal plant spices from the *Ocimum* plant, as they contained rich flavour and scent, mostly due to the presence of essential oils [47]. Subsequently, another study surveyed different individuals on creating nutritional recipes in treating malnutrition using medicinal plants, in which *O. gratissimum* was indicated as one of the most used plants due to its presence of minerals such as calcium, sodium, iron and zinc [76,77].

*O. gratissimum* leaves are a good source of food nutrients. Various analyses, including proximate and elemental analysis, identified the presence of different minerals [74,75,78]. They screened and investigated acetone, methanol, and aqueous extracts, which showed the presence of lipids, iodine, carbohydrates, crude fibres, fat, protein, sodium, potassium, zinc, iron, calcium, manganese, and magnesium, including ash [74,75,76,77,78]. Furthermore, the presence of crude fibre was linked to the potential to lower serum cholesterol levels and with therapeutic activities such as antihypertensive, constipation, and anticancerous effects [75]. Additional minerals identified in the leaf extracts included lower concentrations of lead and nitrogen [74,78].

Additional bioactive compounds identified from the *O. gratissimum* plant include vitamins, tannins, anthraquinones, terpenoids, flavonoids, glycosides, polyphenols, xanthoproteics, resins, steroids, triterpenes, fatty acids, esters, alcohols and oils. Many analysis techniques have been deployed to assess the presence of bioactive compounds, including the use of spectrophotometric assessments, gas and liquid chromatography, flame ionisation detection, and qualitative and quantitative phytochemical screening [77,79,80,81,82,83,84,85].

## 4. Bioactive Compounds of *Ocimum gratissimum* with Molecular Structures

*O. gratissimum* have a profusion of various bioactive compounds which have been extracted from the whole plant, including roots, stems or bark, leaves, and branches. For instance, in a quantitative analysis study, ref. [79] show the presence of various polyphenol bioactive compounds such as tannins, phenols and alkaloids extracted from a leaf extract using hot water as a concentration liquid. The results show the presence of total phenolics (8.47 ± 1.33 mg/g GAE), total tannins (7.84 ± 0.13 mg/g TAE) and total alkaloids at 2.70 ± 0.05 mg/g TAE. In comparison, another study using methanol and hydro methanolic leaf extracts showed total flavonoids [(methanol 151.90 μg/QE mg^−1^; hydromethanolic 15.77 μg/QE mg^−1^)], and total phenols [(methanol 63.05 μg/GAE mg^−1^; hydromethanolic 74.59 μg/GAE mg^−1^)], demonstrating the presence of various polyphenols [86]. Furthermore, such studies’ results demonstrate that the aqueous leaf extracts have higher concentrations of phenolics compared to tannins [79].

Subsequently, in other studies, the presence of these bioactive compounds, such as alkaloids, terpenes, phenols, flavonoids and tannins, were indicated in leaf extracts [29,45,79,87]. Furthermore, [86] conducted an in vitro phytochemical analysis of the aerial portion of the *O. gratissimum* using methanol and hydro-methanol as extracting agents through gas chromatography, and the results presented 51 active oil constituents [86]. These constituents include linalool (32.2%), 1,8-cineole (15.57%), geraniol (14.7%), and epi-a-cadinol (5.5%) [86].

Moreover, a recent review study conducted by [38] indicates that *O. gratissimum*‘s main source of phytochemical compounds is essential oils, which account for about 80% of the total discovered compounds from the whole plant [32]. The review further indicates that the main constituents are eugenol and thymol, which are mostly extracted from the stem and leaves of *O. gratissimum* [38,88]. In another study, an aqueous leaf extract was analysed and used to extract thymol compounds using the voltametric technique [72]. Thymol bioactive compounds were profusely extracted. Thymol is an essential bioactive compound responsible for various pharmacological activities, such as antioxidant, anti-inflammatory, antiseptic, antifungal, anticancerous, immune-regulatory functions and antimicrobial properties [80,89]. In another review study, various roots of *Ocimum* species, including *O. gratissimum*, were examined. The study identified the presence of rosmarinic acid from the root extracts as the main component of the polyphenol constituents [42]. Rosmarinic acid is an essential secondary metabolite which has medicinal therapeutic activities such as antibacterial, antifungal, and antiviral [42,90].

Moreover, a study conducted by [36], using the ethanol extract, found that the presence of flavonoid compound fraction alleviates the effects of oxidative stress and inflammation in male Wistar rats. Furthermore, the active secondary metabolites such as flavonoids, thymol, terpenoids, and eugenol were associated with the ability to block the synthesis of prostaglandins by first inhibiting the activity of cyclooxygenase enzymes and thereafter preventing fever [36,91]. In a later review study of *O. gratissimum*, various polyphenolic secondary metabolite constituents, such as tannins, phenolics, and flavonoids, were highlighted to have enzymatic inhibitory effects on enzyme activities and functions [80]. Moreover, these claims were investigated in a study by [92], which assessed the aqueous extract against penile and testicular tissues and discovered that the presence of these secondary metabolites, including polyphenols, contains inhibitory properties against the following enzymes: angiotensin I-converting enzyme (ACE), acetylcholinesterase (AChE), phosphodiesterase (PDE) and arginase [32,92].

In the following, the chemical structures and functions of each bioactive constituent are provided.

### 4.1. Vitamins

**Figure 1:**
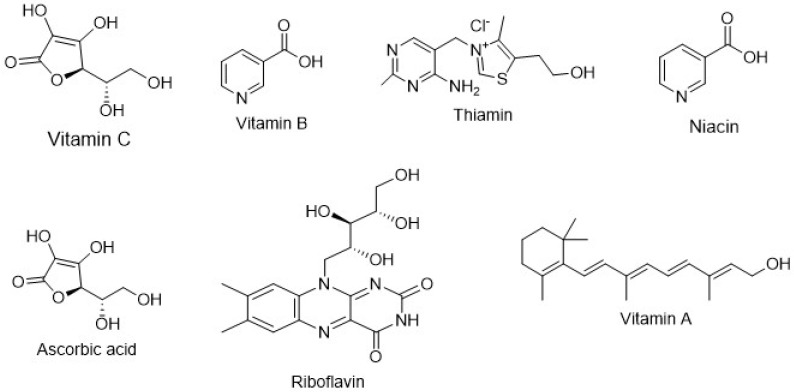
Chemical structures of vitamin constituents in *O. gratissimum*.

*O. gratissimum* is also a source of vitamins. In [3], it is highlighted that vitamins are important for providing nutritional value and other therapeutic effects [3]. Therapeutic effects associated with vitamins found in *O. gratissimum* include antioxidant activity that help improve the immune system, as well as effects on vision and reproduction [78]. Additionally, the presence of vitamin A is associated with improvement of the respiratory functions [93]. The presence of vitamins in *O. gratissimum* has been investigated in various studies such as [78,94,95]. The identified vitamins include ascorbic acid, niacin, thiamin, and riboflavin; see Figure 1 for chemical structures [94,96]. Vitamins A, C and E were reported to be present in the *O. gratissimum* leaf [75,78].

### 4.2. Alkaloids

**Figure 2:**
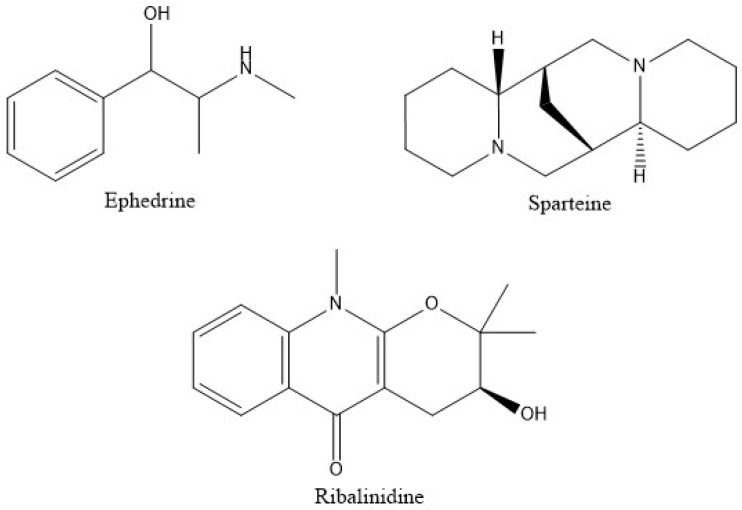
Chemical structures of alkaloids in *O. gratissimum*.

Alkaloids, bioactive compounds, were identified in multiple studies [6,45,74,79,97]. Alkaloids are associated with various therapeutic activities such as anti-inflammation, neurogenerative activities and antioxidants [45,79]. *O. gratissimum*-identified alkaloid constituents include ephedrine [84], while ribalinidine and spartiene presence was noted in some of the studies [60,84,98,99]. Figure 2 shows the chemical structures of alkaloid constituents.

### 4.3. Polyphenols

**Figure 3:**
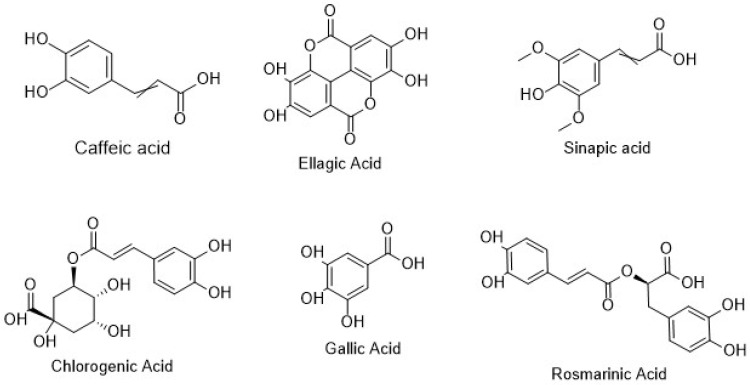
Chemical structures of phenols acids in *O. gratissimum*.

**Figure 4:**
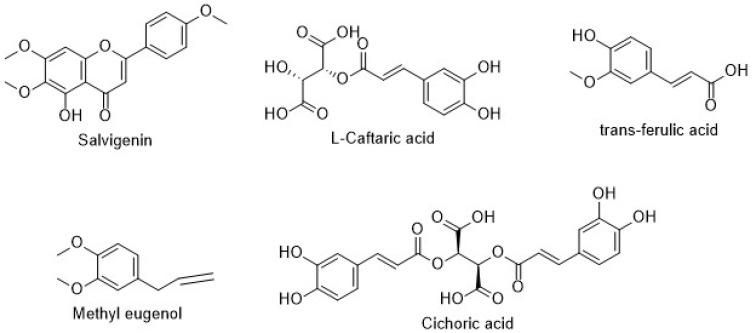
Chemical structures of phenolics in *O. gratissimum*.

*O. gratissimum* is a medicinal plant that is rich in polyphenols. It contains many bioactive compounds such as phenolic acids, phenols, flavonoids, tannins, and resins. Different therapeutic properties and functions of these polyphenols have been investigated. It has also been discovered that the presence of polyphenol bioactive compounds in *O. gratissimum* makes the plant less aversive and more effective in its therapeutic activities when treating various ailments [75,79,100]. Moreover, the presence of polyphenols makes the *O. gratissimum* a good source of antioxidants, as well as making it anti-inflammatory [61,75,79,101]. The presence of the various phenolic acids and phenols was identified in the *O. gratissimum* plant, including caffeic acid, chlorogenic acid, ellagic acid, gallic acid, Rosmarinic acid and sinapic acid; see Figure 3 for their chemical structures [32,62,99]. Additionally, salvigenin and methyl eugenol were identified [32,75]. Furthermore, L-caftaric acid, trans-ferulic acid [36,62,102] and L-chicoric acid presence was indicated in a study by [100]. Figure 4 illustrates phenolics chemical structures.

**Figure 5:**
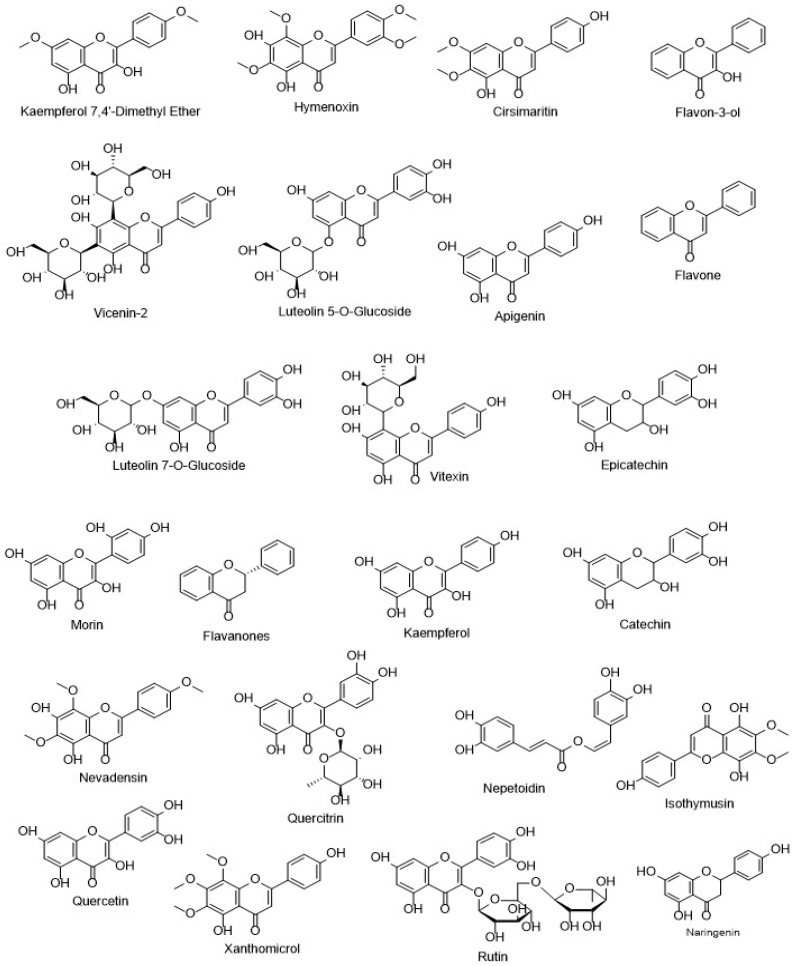
Chemical structures of flavonoids in *O. gratissimum*.

Other forms of polyphenols, the flavonoids, are present in *O. gratissimum.* Flavonoid constituents identified in *O. gratissimum* include Flavan-3-ol [84], Apigenin, Catechin, Epicatechin, Flavone, Flavanones, Kaempferol, Morin, Isothymusin, Naringenin [103], Luteolin, Quercetin, Rutin, Nepetoidin A, Quercitrin, Xanthomicrol, Nevadensin, Lutelion-7-O-Glucoside, Lutelion-5-O-Glucoside, Vicenin-2 (also known as Apigenin 6,8-di-C-glucoside), Cirsimaritin, Hymenoxin, and Kaempferol 7,4′-dimethyl [80,104,105]. Figure 5 illustrates the flavonoids’ chemical structures, and ethers were also quantified [62,75,84,99] alongside vitexin compounds [32].

### 4.4. Triterpenes and Steroids

**Figure 6:**
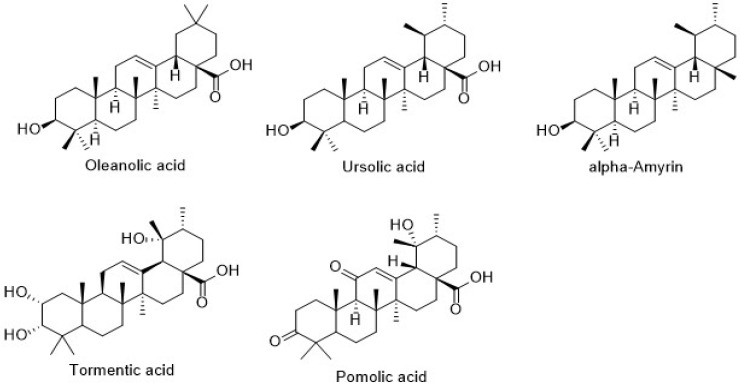
Chemical structures of triterpenes and steroids in *O. gratissimum*.

Triterpenes and steroids were identified in *O. gratissimum*, including oleanic acid [97,106], ursolic acid, tormentic acid, and pomolic acid; see Figure 6 [32]. Moreover, α-amyrim and 4,22 stigmastadiene-3-one presence was identified in a gas chromatography study conducted by [49,107]. Furthermore, triterpenes were discovered to have therapeutic benefits associated with antidiabetic [32], anti-viral properties [106] and insecticidal activities [87]. Figure 6 illustrates the chemical structures of triterpenes and steroid constituents.

### 4.5. Fatty Acids and Esthers

**Figure 7:**
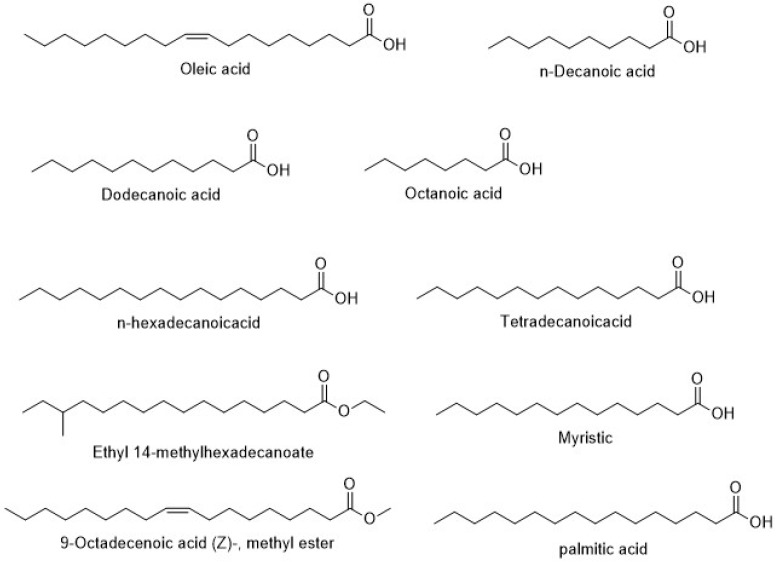
Chemical structures of fatty acids and esters in *O. gratissimum*.

Fatty acids, bioactive compounds in *O. gratissimum*, are associated with their impact on lipid metabolism, which was identified to improve the serum lipid profile of male albino rats [108]. Various metabolites associated with fatty acids were identified in a recent study and exhibit antibacterial properties [49]. The fatty acid constituents include: n-Decanoic acid, Tetradecanoic acid, Dodecanoic acid, n-Hexadecanoic acid, 9-Octadecenoic acid (Z)-, methyl ester, Oleic Acid, Ethyl 14-methyl-hexadecanoate, Octanoic acid, Myristic and palmitic acid, as illustrated in Figure 7 [45,49,85]. Figure 7 illustrates their chemical structures.

### 4.6. Alcohols

**Figure 8:**
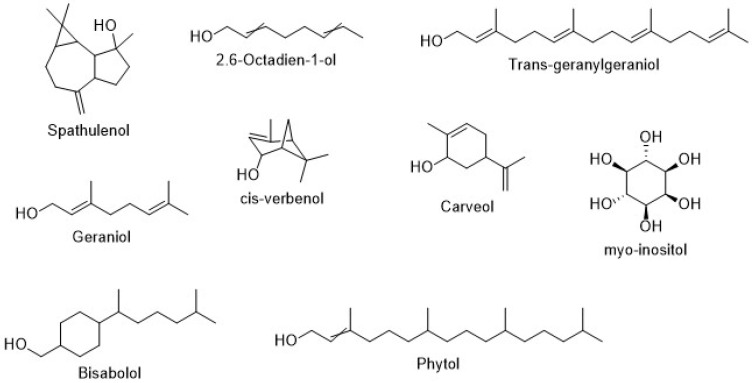
Chemical structures of alcohols in *O. gratissimum*.

*The* presence of alcohol in *O. gratissimum* has been identified to have a variety of therapeutic benefits, including acting as a reducing agent [100]. Some of the therapeutic benefits of alcohol from *O. gratissimum* include antimicrobial activities and anti-inflammatory and insect-repellent properties [32]. *O. gratissimum*-identified alcohol constituents are carveol, cis-verbenol, 2,6-octadien-1-ol, and myo-inostol; see Figure 8 [45,85,107]. Furthermore, ref. [45] indicates the presence of phytol and trans-geranylgeraniol [45,109]. Other alcohol constituents include geraniol and Spathulenol [32] and bisabolol [110].

### 4.7. Ketones and Aldehydes

**Figure 9:**
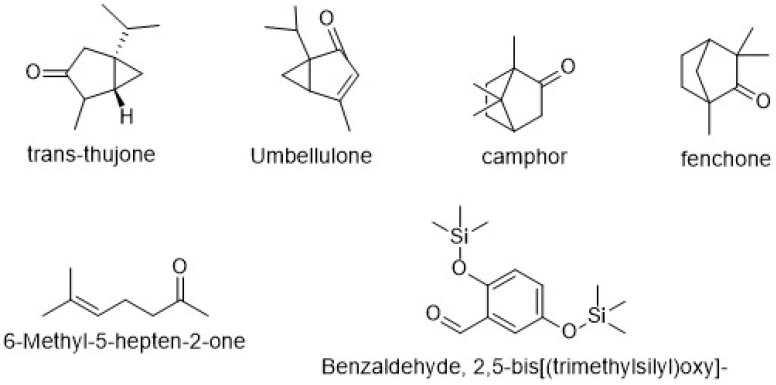
Chemical structures of ketones in *O. gratissimum*.

**Figure 10:**
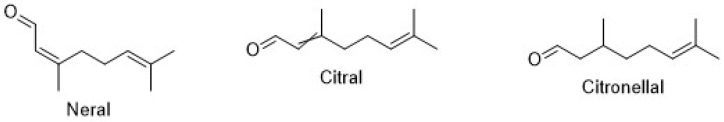
Chemical structures of Aldehydes in *O. gratissimum*.

In a recent review study, a variety of ketones were identified to be present in *O. gratissimum* plant; the identified ketones include trans-thujone, umbellulone, camphor, and fenchone—see Figure 9 [32]. Additionally, 6-Methyl-5-hepten-2-one and Benzaldehyde, 2.5-bis-(trimethylsilyl)-oxy]- presence was found [81,85,111]. Low concentrations of aldehydes were found in the plant, and minor constituents found include cintronellal, citral and neral, see Figure 10 [32]. Figure 9 and Figure 10 below shows chemical structures of ketones and aldehydes, respectively.

### 4.8. Oils and Essential Oils

**Figure 11:**
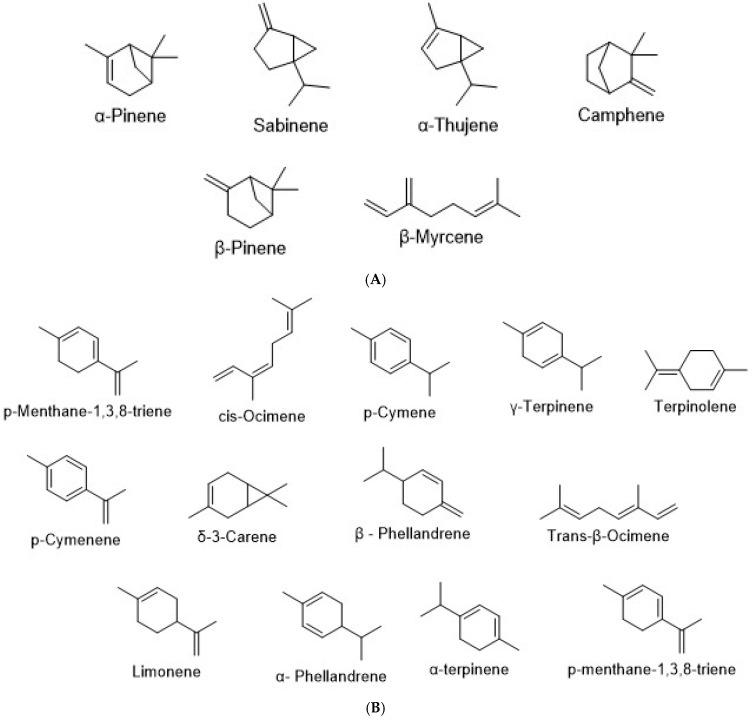
(**A**) Chemical structures of hydrocarbonated monoterpenes in *O. gratissimum*. (**B**) Chemical structures of hydrocarbonated monoterpenes in *O. gratissimum*.

**Figure 12:**
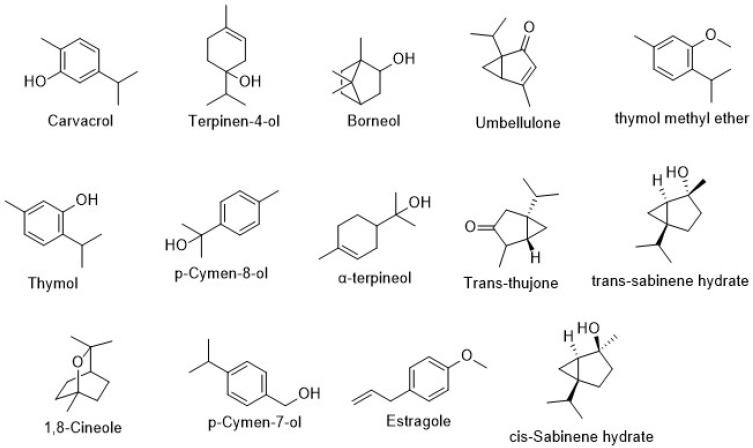
Chemical structures of oxygenated monoterpenes in *O. gratissimum*.

**Figure 13:**
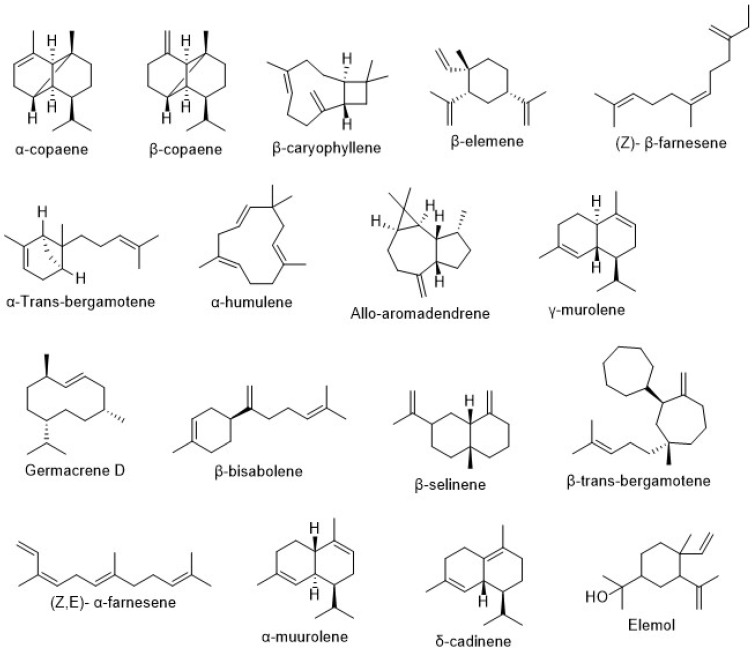
Chemical structures of hydrocarbonated sesquiterpenes in *O. gratissimum*.

**Figure 14:**
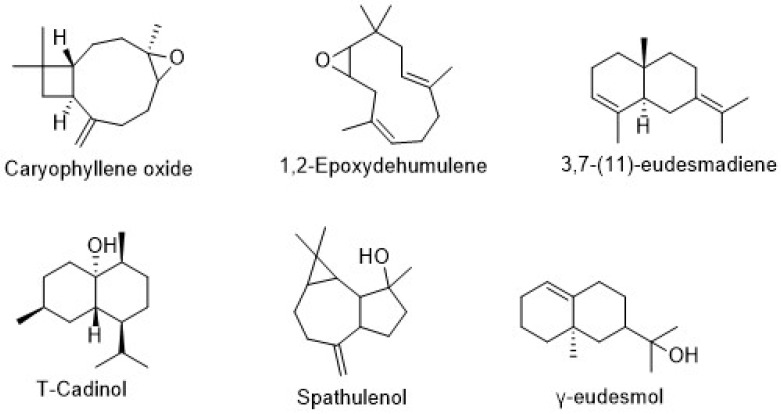
Chemical structures of oxygenated sesquiterpenes in *O. gratissimum*.

The presence of essential oil in the *O. gratissimum* gives the plant a strong aromatic scent [32]. *O. gratissimus’* most attributed essential oils include thymol and eugenol [36,88,112,113]. Several studies highlight the pharmacological benefits of thymol and eugenol, including antioxidants, which demonstrate high free radical scavenging potential against different radicals and inhibit oxidative stress in cellular damage [75,79,100]. Additionally, the identified essential oils from *O. gratissimum* can be classified into 4 types of groups, namely: hydrocarbonated monoterpenes (Figure 11A,B), oxygenated monoterpenes (Figure 12), hydrocarbonated sesquiterpenes (Figure 13) and oxygenated sesquiterpenes (Figure 14) [23,47,57,114].

Hydrocarbonated monoterpenes identified from the *O. gratissimum* plant include: camphene, α-thujene, α-pinene, β-pinene, sabinene, β-myrcene (Figure 11A) and α- and β-phellandrene, δ-3-carene, limonene, α-terpinene, p-cymene, trans-β-ocimene and cis-ocimene, γ-terpinene, terpinolene, p-cymenene, and p-menthane-1,3,8-triene, (Figure 11B) [32,47,97,114].

Moreover, the oxygenated monoterpenes identified in *O. gratissimum* are associated with various pharmacological activities such as anti-inflammatory, antioxidant, anti-repellent and antinociceptive activities [32,36,79]. Oxygenated monoterpenes are described as complex compounds as they are characterised by oxygen-containing chemical structures, such as those similar to alcohol, ethers, phenol, and aldehydes [115]. Additional identified oxygenated monoterpenes of *O. gratissimum* include: 1,8-cineole, cis-sabinene hydrate, linalool, trans-sabinene hydrate, trans-thujone, citronellal, umbellulone, borneol, terpinen-4-ol, p-cymen-8-ol, α-terpineol (also referred to as alpha-terpineol), thymol methyl ether, estragole, p-cymen-7-ol, thymol and carvacrol; see Figure 12 [36,79,114].

Several hydrocarbonated sesquiterpenes are indicated to be present in the *O. gratissimum* plant. Hydrocarbonated sesquiterpenes are associated with various therapeutic benefits such as gastroprotective, immune-modulatory, antimicrobial, antioxidant, and hepatoprotective effects [36,79,97]. Some of the identified hydrocarbonated sesquiterpenes includes: α-copaene, β-elemene, β-copaene, β-caryophyllene, α-Trans-bergamotene, (Z)- β-farnesene, α-humulene, Allo-aromadendrene, γ-murolene, Germacrene D, β-trans-bergamotene, β-selinene, β-bisabolene, (Z,E)- α-farnesene, α-muurolene, δ-cadinene, and Elemol; Figure 13 illustrates their chemical structures [6,7,32,116].

Ref. [117] highlight the essence of oxygenated sesquiterpenes in a molecular docking study, in which they found the presence of the oxygenated sesquiterpene constituent compound caryophyllene oxide. The researchers discovered that caryophyllene oxide interacts and inhibits lipoxygenase and other inflammatory cytokines, such as interleukin-6 (IL-6), interleukin-1 (IL-1), and tumour necrosis factor-α (TNF-α) [117]. Subsequently, Zakariyah and colleagues highlight the therapeutic benefits of antibacterial agents in a study involving *Nigrospora oryzae* [49]. The identified oxygenated sesquiterpenes of *O. gratissimum* include: Caryophyllene oxide, 1,2-epoxydehumulene, 3,7-(11)-eudesmadiene, Spathulenol, T-cadinol and γ-eudesmol; see Figure 14 [5,7,9,32,33,52,118].

## 5. Structure–Function Activity Relationship

*O. gratissimum* contains multiple bioactive compounds formed from different structure groups such as essential oils and volatile and non-volatile secondary constituent compounds. The structure–activity relationship (SAR) of different bioactive compounds in *O. gratissimum* is determined by their functional groups and structural designs, which then determines the structure’s potency as antioxidant, cognitive enhancing, antimicrobial, anti-inflammatory agents [105]. The structures, functional groups, and designs of these bioactive compound constituents explain how *O. gratissimum* utilises complex phytochemical networks to interact with and target critical biological targets [117]. *O. gratissimum* has multiple SARs, such as phenolic compounds and antioxidants SAR, flavonoid SAR and membrane protection, antimicrobial and anti-inflammatory SAR, cognitive enhancement and phenols SAR. Table 1 represents the SAR of *O. gratissimum* bioactive compounds, the targeted biological sites and activity outcomes.

Furthermore, some of the *O. gratissimum* SAR insights are essential indicators of the role bioactive compounds play in regulating pharmacological activities. Some of the key insights include antioxidants’ efficiency, which relates to free radicals scavenging activities that depend on phenolic rings, such as alkyl groups, which act as electronic donors to boost antioxidant activity potency. -COOH has electron-withdrawing abilities [105]. Subsequently, some SARs are linked to modulating various pathways, such as suppressing survival signals linked to extracellular signal-regulated signal (ERK) in cancer cells and activating protective pathways and nuclear factor erythroid 2-related factor (NRF-2) in healthy cells [119,120]. Moreover, additional *O. gratissimum* SAR includes dual pathway inhibition, which involves constituents, such as thymol and eugenol, that inhibit lipoxygenase and cyclooxygenase pathways by preventing the effects of respiratory problems linked to bronchoconstriction [119].

**Table 1:** Structural–function activity relationship of *O. gratissimum* bioactive compounds.

Functional Group	Bioactive Constituent	Associated Mechanism	Outcomes	References
Phenolic Hydroxyl (-OH) groups	Eugenol and Thymol	Act as hydrogen donors to fight and neutralise free radicals	Potent antioxidant activities.	[105]
Hydroxyl-mediated	Eugenol and Luteolin	Changes membrane fatty acids and affects the cytoplasmic membrane and ATP leakage	Potent antibacterial activity	[60,121]
Alkyl-Hydroxyl group	Thymol and Carvacrol	Stabilises phenoxy radicals by donating electrons, subsequently increasing electron density	Increased antioxidant potency	[105]
Methoxy mediation	Methyl-eugenol and Eugenol	Stabilises free radicals and increases hydrogen-donating potential	Increases antioxidant capacity	[105]
Catechol structures group	Luteolin and Apigenin	Mediates and promotes electron donation for free radicals	Enhances neuroprotection and strengthens membrane protection	[122]
Beta-sheet binding structure	Rosmarinic acid	Involved in directly binding to the beta sheet structure of amyloid-beta oligomers and fibrils, which inhibits alpha–beta aggregation	Anti-Alzheimer’s effects	[105,123]
Phenolic rings	Thymol and Eugenol	Blocks prostaglandin synthesis by binding to the COX	Analgesic and anti-inflammatory effects	[117]
Nucleophilic-Cysteines interaction	Rosmarinic acid and Eugenol	Triggers NRF2 release by interacting with nucleophilic cysteine on the KEAP1 protein	Activates redox homeostasis	[123,124]
Sesquiterpene structure groups	Caryophyllene	Mediates specific agonist for the CB2 endocannabinoid receptors	Immune modulator and anti-analgesic effects	[121,125]

## 6. Pharmacological Activities

*O. gratissimum*’s pharmacological activities have been extensively researched and well-documented by different researchers and authors. The pharmacological activities of *O. gratissimum* are possible due to the vast availability of bioactive compounds such as essential oils, polyphenols, alcohols, ketones, aldehydes, fatty acids, esters, alkaloids and vitamins. These bioactive compounds are responsible for making *O. gratissimum* a medicinal plant with a variety of therapeutic properties that function in treating different ailments. *O. gratissimum* therapeutic properties include antioxidants [32,45,74,75], neuroprotection [36], enzyme effects [79,108], anti-inflammatory activities [36,39,60,100,126], anticancer, antidiabetic, antidiarrheal, antimicrobial and antinociceptive effects [32,45,74,79,87,117] amongst others.

The pharmacological properties are reported in different studies, which include in vivo, in vitro, comparative and systematic reviews, survey studies, and clinical trials [34,38,60,70,81,96,108,113,127,128]. Table 2 below indicates and summarises recent pharmacological studies of *O. gratissimum*.

### 6.1. Pharmacological Mechanisms

Most medicinal plants exhibit a variety of pharmacological properties because they are rich in bioactive compounds capable of treating various ailments [133,134]. The presence of bioactive compounds enables medicinal plants to possess unique traits, such as therapeutic effects, mediated by biochemical and pharmacological mechanisms [135]. These compounds influence physiological and molecular processes to mediate diseases. *O. gratissimum* has multiple pharmacological mechanisms, including nootropic, anti-convulsion, antioxidant, antimicrobial, anti-inflammatory, anti-antigenic, anticancerous, and anti-plasmodial effects.

**Figure 15:**
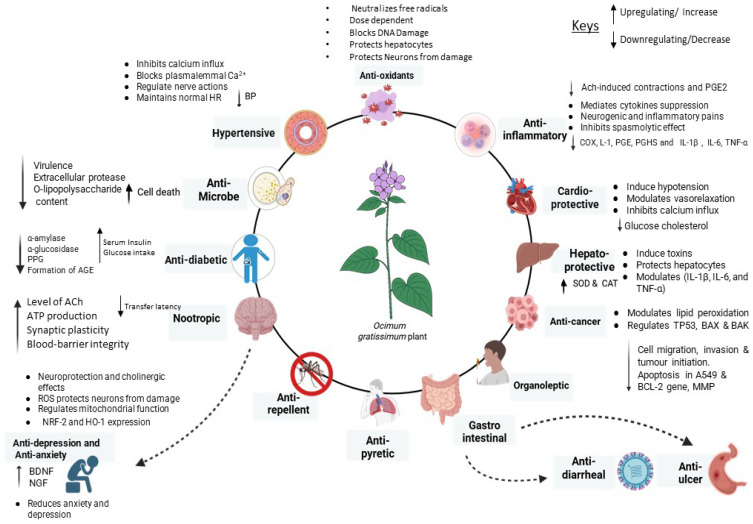
Comprehensive molecular mechanism of the pharmacological actions of *O. gratissimum*. Abbreviations: Ach—Acetylcholine; PGE2—prostagladins E2; COX—cyclooxygenase; PGHS—prostaglandin H; L-1—lipoxygenase; PGE—prostagladins; IL-1β—interleukin 1-β; IL-6—interleukin-6; IL-1α—interleukin 1-α; TP53—tumour protein p53; BAX—X protein gene; BAK—BCL2 Antagonist/Killer 1; TNF-α—tumour necrosis factor-α; BCL-2—B-cell lymphoma-2; MMP—matrix metalloproteases; SOD—Superoxide dismutase; CAT—Catalase; BDNF—brain-derived neurotrophic factor; NRF-2—nuclear factor erythroid 2-FVGF-related factor-2; HO-1—Heme oxygenase-1; NGF—Nerve growth factor; ATP—Adenosine triphosphate; AGE—advanced glycation end products; BP—blood pressure; HR—heart rate; and DNA—deoxyribonucleic acid. Figure created using BioRender.com.

*O. gratissimum* pharmacological effects are controlled by different cellular signalling pathways. Some of the molecular mechanisms highlighted in this review include COX, LP-1, PGE, PGHS, and IL-1β, IL-6, and TNF-α signalling pathways, as well as TP53, BAX and BAK modulations, and PGE2 and Ach. The highlighted signalling pathways are shared in various therapeutic and pharmacological activities. Figure 15 provides a comprehensive overview of the molecular mechanisms underlying the pharmacological and biological effects of *O. gratissimum*.

#### 6.1.1. Antioxidants

*O. gratissimum* is rich in naturally derived compounds, including antioxidants. Antioxidants are synthetically produced compounds that play a role in inhibiting the reactions of free radicals, which would cause oxidative stress and degenerative diseases [28]. *O. gratissimum* antioxidants are key fixtures and contributors to an array of pharmacological activities. The underlying antioxidant therapeutic activities are exerted by different bioactive compounds. The presence of essential oils, thymol, flavonoids, terpenoids and alkaloids collaboratively provides *O. gratissimum* with antioxidant therapeutic effects. Other researchers, such as [60,77], indicate rutin and saponins as some of the bioactive compounds that contribute to the antioxidant pharmacological properties. For instance, *O. gratissimum* can neutralise free radicals. The presence of thymol in *O. gratissimum* mediates the ability to facilitate the scavenging molecular cycle. In one of the studies, it highlights the ability of *O. gratissimum* to scavenge DPPH; the study results confirmed the DPPH scavenging half-maximal concentration value to be 100.00 ± 0.00 µg/mL [22]. Another scavenging concentration was recorded at 4.84 ± 0.34 µmol TE 9^−1^ FW [22], while 88.8 ± 0.63 w/v% is recorded in [79].

Subsequently, another antioxidant pharmacological potential of *O. gratissimum* is reducing power, which includes donation of electrons to reduce oxidised mediators by converting Fe^3+^ to Fe^2+^ [79]. A study investigated the reducing power of *O. gratissimum* aqueous leaves at different concentrations (3–21 w/v%). The results ranged from 0.68 ± 0.01 to 1.67 ± 0.11 (from low to high concentration). Moreover, ref. [70,136] conducted a reducing power assay using various concentration levels (0.2–1 mg/mL) with the reducing power inhibition results of 0.2–0.4 nm [70]. In addition, ref. [113] conducted an investigation of *O. gratissimum* leaves and roots and conducted the FRAP test; the leaves’ results were 23.89 ± 00.73 mol TE g^−1^ FW, while roots resulted in 22.41 ± 1.05 mol TE g^−1^ FW. FRAP was conducted on aqueous extracts (2.94 ± 0.03 mg AAE/g) [113]. The previous studies’ results highlight the potential of *O. gratissimum* to convert free radicals to be less aversive and the potential to reduce oxidised mediators.

#### 6.1.2. Cognitive and Memory Enhancement Potential: Nootropic Properties

In a review, ref. [60] explore the various effects of *O. gratissimum* on memory improvement and neuroprotective potential, which were investigated in various animals. According to a recent study, ref. [137] found that *O. gratissimum* leaves extract exhibited memory and learning enhancement effects, as evidenced by the animal demonstrating decreased transfer latency and improved step-down latency [60,138]. Furthermore, the review further explored the exposure of *O. gratissimum* in animal models, and the findings indicated that the plant has nootropic properties and potential in improving working memory. This is similar to a study conducted by [139], which highlights an improvement in working memory. Other studies showed improved development in spatial memory [60,129,138], and one other later study indicated an increase in long-term memory consolidation [138].

**Figure 16:**
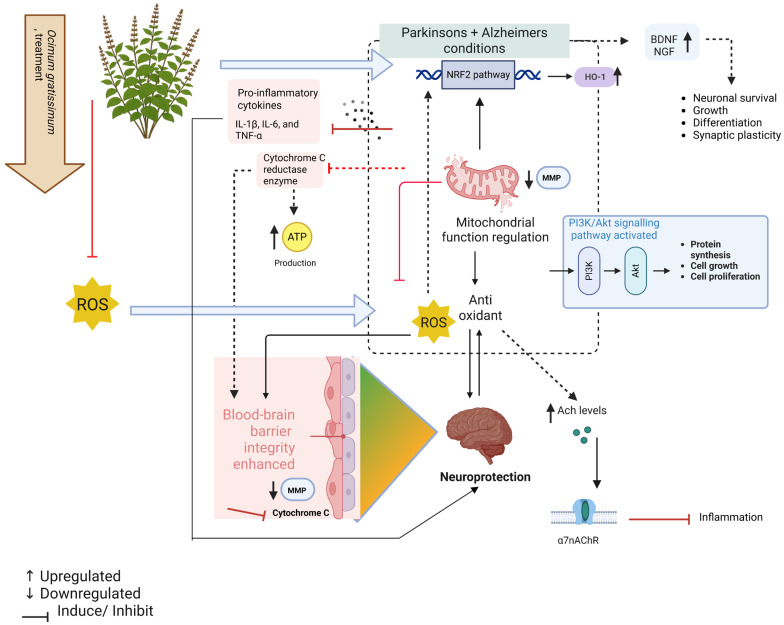
Overview of molecular mechanisms of cognitive enhancement of *O. gratissimum*. Abbreviations: Ach—Acetylcholine; IL-1β—interleukin 1-β; IL-6—interleukin-6; IL-1α—interleukin 1-α; TP53—tumour protein p53; BAX—X protein gene; BAK—BCL2 Antagonist/Killer 1; TNF-α—tumour necrosis factor-α; BCL-2—derived neurotrophic factor; NRF-2—nuclear factor erythroid 2-related factor-2; HO-1—Heme oxygenase-1; NGF—Nerve growth factor; ATP—Adenosine triphosphate; AGE—advanced glycation end products; BP—blood pressure; HR—heart rate; and DNA—deoxyribonucleic acid. Figure created using BioRender.com.

*O. gratissimum* has neuroprotective effects, which are highlighted in a few recent studies. For instance, Aduyi and colleagues highlight the ability of *O. gratissimum* on the impact of oxidative stress effects and inhibition of neuroinflammation [8,60,140]. Meanwhile, a different study explored the exposure of the *O. gratissimum* ethanol leaf extract against focal ischemia and reperfusion in male Wistar rats, which revealed the plant’s potential to impact and regulate the neurotropic factors, such as enhancing synaptic plasticity, regulation of mitochondrial function, enhancement of blood-barrier integrity, and gene expression potential, which subsequently activates the nuclear factor erythroid 2-related factor (NRF2) signalling pathway; ref. [129]. Figure 16 illustrates the molecular mechanisms of action from these studies.

#### 6.1.3. Antimicrobial

In a review, ref. [38] highlights the antibacterial properties of *O. gratissimum* and the plant’s ability to inhibit microbial pathogens, including common bacterial strains such as *Salmonella typhimurium*, *Escherichia coli* (*E. coli*), and *Staphylococcus aureus*, which are associated with causing diarrhoea [38]. The review further highlights the assessment of *O. gratissimum* water leaf and indicated a minimum concentration variation of about 0.001–0.1% across the bacterial strains. In another study, ref. [85] investigated the antibacterial properties of the plant against various bacterial strains such as *Escherichia coli*, *Salmonella typhi*, *Proteus vulgaris*, *Shigella flexneri*, *Citrobacter freundii*, and *Morganella morganii*. The bacterial strains are associated with causing gastroenteritis. The study assessed the various *O. gratissimum* extracts, including ethanol extract, and compared their potency against bacterial inhibition. From this study, it is highlighted that ethanol extract demonstrated the highest inhibition zone of 29.67 ± 0.33 mm against *E.coli* [85]. Similarly, an *O. gratissimum* ethanol extract was studied and found to be more susceptible against the following bacteria: *Staphylococcus aureus*, *Escherichia coli*, *Klebsiella pneumoniae* and *Candida albicans* [141]. Additionally, the authors emphasised that the antimicrobial properties of *O. gratissimum* are expressed by the available abundance of bioactive compounds such as terpenoids, steroids, flavonoids, alkanoids, phlabotannins and tannins [141].

#### 6.1.4. Anti-Inflammatory

Refs. [3,142] highlight that inflammation is a critical process which is needed to maintain and protect an organism’s body against pathogens [3,142]. *O. gratissimum* has been researched and documented to contain anti-inflammatory properties, which occur through various mechanisms. For example, some of *O. gratissimus’* pharmacological activities in inflammatory management include inhibition of inflammatory mediators, regulation of inflammatory cytokines, and pain management. The anti-inflammatory mechanisms are promoted by some bioactive compounds, such as rosmarinic acid, essential oils, phenolics, flavonoids, and eugenol compounds [112,143].

For instance, *O. gratissimum* bioactive compounds, such as essential oils, play a major role in regulating and promoting essential inflammatory mediators and biomarkers, including the inhibition of cyclooxygenase (COX) through the inhibition of two enzymes involved in inflammation production, namely prostaglandin H synthase (PGHS) and lipoxygenase (LP-1) [110,144]. The study inhibition concentration values were measured to be at 125 µ/mL and 144µ/mL repetitively for the enzymes [110,144]. In a review study, ref. [60] unpack various studies highlighting the role of flavonoid and eugenol from *O. gratissimum*, which contain the potential to inhibit pro-inflammatory cytokines, including interleukin 1-β (IL-1β), interleukin-6 (IL-6) and tumour necrosis factor-α (TNF-α) [32,60,143]. Interleukins 1-8 and IL-6 were noted to be reduced in [5]; see Figure 16. In a similar study, the essential oil constituent of *O. gratissimum* leaf extract was found to decrease the baseline concentration of prostaglandin E2 (PGE2) by 46% when adjusted to a concentration level of 5–20 µg/mL [5].

**Figure 17:**
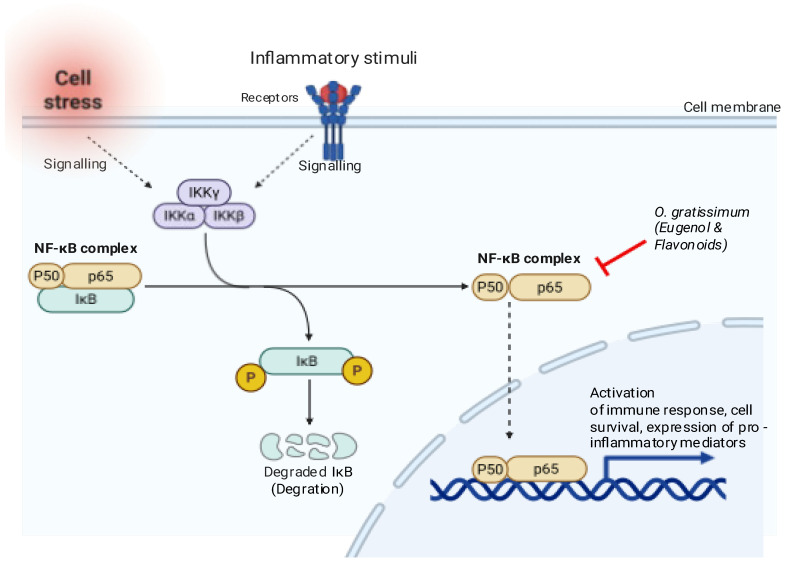
NF-kB activation as a target for *O. gratissimum* eugenol and flavonoids. Cell stress, inflammation mediators, and growth factors. TNF-α, IL-1, and IL-6 activate NF-κB. Additionally, bioactive compounds, eugenol and flavonoids, are shown to inhibit the activity of IKK-*y*, α and β, which subsequently leads to suppression of the NF-kB activation, and activate immune response towards inflammatory cell stress. Inhibition is represented by the red line. Abbreviations: IL-1β—interleukin 1-β; IL-6—interleukin-6; IL-1α—interleukin 1-α; TNF-α—tumour necrosis factor-α; Nuclear factor kappa B (NF-Kb); inhibitory molecules of the kinase (IKK). Figure created using BioRender.com.

The identified bioactive compounds, eugenol and flavonoids, contribute to the overall anti-inflammatory molecular mechanisms. Eugenol and flavonoids have the potential to prevent the upregulation of nuclear factor kappa B (NF-kB) in inflammatory conditions [145]. Ref. [145] further highlights that the transcription factor NF-kB modulates the production of pro-inflammatory genes, which are responsible for chemokines and the encoding of cytokines and related enzymes such as COX. The production of pro-inflammatory mediators elevates NF-kB activities to downregulate immune cell activation, which subsequently lowers inflammatory stress signal [145]. Eugenol and flavonoids have the potential to inhibit the production of cytokines. When a chemical cell stressor occurs, it activates NF-kB signal pathways. The therapeutic properties of eugenol and flavonoids collaboratively trigger various inhibitory molecules to inhibit NF-kB signal pathway. The different inhibitory molecules released include IkKα and IkKβ (Figure 17 shows the pathway). When the NF-kB pathway is activated, the NF-kB proteins, such as p65 and p50, undergo phosphorylation, which is later degraded. The inhibitory proteins are deactivated by the IkB kinase complexes. The IkB kinase complex is made up of the following molecules: IKK-α, IKK-β and IKK-*y* [145].

#### 6.1.5. Anti-Analgesic

Some of the anti-inflammatory properties are associated with pain management. *O. gratissimum* plant extracts are linked to the management and treatment of pain and the demonstration of their antinociceptive properties. Various studies, such as those conducted by [60,81,110], have reviewed and detailed the application and molecular mechanisms of *O. gratissimum* and its role as an anti-analgesic applicable medicinal plant [60,81,110].

In a review study, ref. [137] reviewed various later and recent studies that found that the available bioactive compounds, such as eugenol, myrcene and essential oils, have the potential and ability to reduce neuroinflammation in mice studies [146,147,148,149]. Moreover, in a later study, Prabhu and colleagues reviewed *O. gratissimum* aqueous extract, which demonstrated anti-inflammatory properties when subjected to agar-induced tests in male Wistar rats. The study showed that *O. gratissimum* extract has significant similarity to the actions of phenylbutazone [110]. While the anti-inflammatory properties of the *O. gratissimum* are attributed to the availability of bioactive compounds from the plants, *O. gratissimum* is a rich source of bioactive compounds, as indicated and illustrated in Figure 1, Figure 2, Figure 3, Figure 4, Figure 5, Figure 6, Figure 7, Figure 8, Figure 9, Figure 10, Figure 11, Figure 12, Figure 13 and Figure 14. Bioactive compounds that contribute to analgesic effects include eugenol and polyphenols [60], thymol, myrcene [81], β-caryophyllene, rosmaric acid, caffeic acid [84], and phenolic compounds such as flavonoids, tannins, saponins [77,84].

#### 6.1.6. Anticancerous

Cancer is linked to about 9.8 million individual deaths each year worldwide [3]. Several researchers have investigated medicinal plants’ anticancer properties, including the use of *O. gratissimum*. One of the studies details that *O. gratissimum* leaf extract has anticancerous properties, by exhibiting cytotoxicity towards cancerous cells [13]. Furthermore, another study utilising the *O. gratissimum* leaf aqueous extract had an effect in decreasing cell apoptosis with the concentration value of 0.2 mg/mL when treating human hepatocellular carcinoma cell lines [119]. Moreover, another study adds that cell apoptosis occurred as the available bioactive compounds in *O. gratissimum* increased oxidative stress and had an effect on mitochondrial membrane potential loss [13,119].

Additionally, the pharmacological mechanisms of *O. gratissimum* on chemopreventive effects are mainly attributed to their potent antioxidant activities. For example, *O. gratissimum* phenolic compounds, such as flavonoids, phenols, alkaloids and saponins, contain modulative effects on lipid peroxidative [79]. Lipid peroxidation is responsible for and involved in the development of carcinogenesis. Ref. [150] add that the antioxidant properties of *O. gratissimum*, mediated by bioactive constituents such as 2-1 isopropyl-5-methylphenol, have the potential to eliminate free radicals. The development of free radicals has the effect of causing oxidative damage to DNA, protein structure, and lipids [151]. *O. gratissimum* has demonstrated potential in scavenging free radicals such as DPPH, peroxide and nitric oxide [41,118]. Furthermore, it has been noted that *O. gratissimum* bioactive compounds have potential in reducing oxidative stress, which leads to cell protection against carcinogenesis [79]. Moreover, the development of chronic inflammation is associated with a risk factor for the development of cancer [152]. *O. gratissimum* contains anti-inflammatory properties which are capable of inhibiting and modulating lipid peroxidation [79].

The ability to regulate and modulate anticancerous properties is facilitated by several bioactive compounds, such as terpenes and terpenoids [81,99]. Terpenes and terpenoids have the potential to inhibit tumour growth, induce cell apoptosis and exhibit cytotoxic effects towards cancerous cells [153].

*O. gratissimum* has anticancer therapeutic potential mediated through the phosphoinositide-3 kinase/protein kinase B (PI3K/Akt) signalling pathway. The PI3K/Akt signalling pathway is an essential biochemical switch that regulates cell proliferation, survival and growth [154]. Eugenol and flavonoids are key bioactive compounds that attenuate the PI3K/Akt signalling pathway [60]. These bioactive compounds inhibit Akt signalling activation, which subsequently prevents metastatic progression. In addition, activation of PI3K/Akt triggers pro-apoptotic mechanisms. For instance, when PI3K/Akt is suppressed, caspases 3, 8 and 9, which are regulatory enzymes that destroy cancerous cell structures, are activated. This suppression also leads to upregulation of pro-apoptotic genes, including TP53, BAX and BAK, and downregulation of anti-apoptotic genes, such as BCL-xL and BCL-2 [60,155].

#### 6.1.7. Anti-Hypertensive and Vasorelaxant

Eugenol, essential oils, and rutin are some of the key bioactive compounds that contribute to the pharmacological activities *of O. gratissimum*, including antihypertensive and vasorelaxant effects [110,156,157]. The pharmacological activities of antihypertensives are associated with vasodilation. Vasodilatation is a body process involving the relaxation of the smooth muscle in the vascular wall. Subsequently, vasodilation is an essential response of smooth muscles to maintain homeostasis by distributing and facilitating oxygen to tissues as the demand grows [158]. *O. gratissimum* antihypertensive activity was researched in a study [157]. The study investigated whole-plant extracts, including leaves, stems, and flowers, by extracting bioactive compounds with aqueous extracts and treating hypertensive rats with the plant extracts. The study further indicates vasorelaxant effects in spontaneously hypertensive rats, lowering blood pressure and heart rate. Additionally, angiotensin-converting enzyme (ACE) activity was inhibited, and ACE levels were reported to decrease [157].

Moreover, vasodilation is a key effector of hypertensive mechanisms. A review by [60] highlights the recent and later studies, which were conducted in vivo. The review highlights one study that utilised *O. gratissimum* leaf extract at 1–20 mg/kg dose in mice, which has shown antihypertensive potential by exhibiting a dose-dependent decline in the mice, further increased mean aortic pressure and reduced heart rate of *O. gratissimum* extract-treated mice [60,159]. Additionally, in another later study, which is highlighted in a review conducted by [110], they highlight the function and role of calcium blockages. The review suggests that the calcium blockages are facilitated by the calcium influx. This potential is demonstrated by a study that utilised *O. gratissimum* essential oil from the aerial portion of the plants as extracts treated on salt hypertensive rats; the study compared the induced extract group and propranolol-treated rats. The *O. gratissimum*-treated rats demonstrated reversible hypotension, which was associated with vasorelaxant effects by removal of the vascular endothelium. Moreover, the study also highlights the effects of reduced calcium-induced contractions and blocking of plasmalemmal calcium influx released from the sarcoplasmic reticulum [156].

#### 6.1.8. Hepatoprotective

*O. gratissimum* has hepatoprotective pharmacological potential. Hepatoprotective effects are plants’ ability to protect the liver by using their bioactive compounds, like phenols and essential oils, which exert protective effects, such as catalase, scavenging oxidative stress and removing toxins from the liver [160]. Some of the *O. gratissimum* hepatoprotective potential has been identified through various studies. For example, one study utilised the *O. gratissimum* leaf extracts by administering the extract to male albino rats induced with carbon tetrachloride (CCl_4_). The study demonstrated dose-dependent hepatoprotective effects of *O. gratissimum*; for instance, the concentrations that showed effects in male albino rats ranged from 200 to 600 mg/kg bw. Hepatoprotective effects observed include inhibition of oxidative stress and repair of liver damage. Observed improved hepato-markers concentrations included aspartate transaminase (AST) (90.3 ± 2.12 IU/L), alanine transaminase (ALT) (35.3 ± 4.34 IU/L), alkaline phosphatase (ALP) (40.2 ± 3.91 IU/L) and total bilirubin (TB) as 0.2–1.3 mg/dL [21].

**Figure 18:**
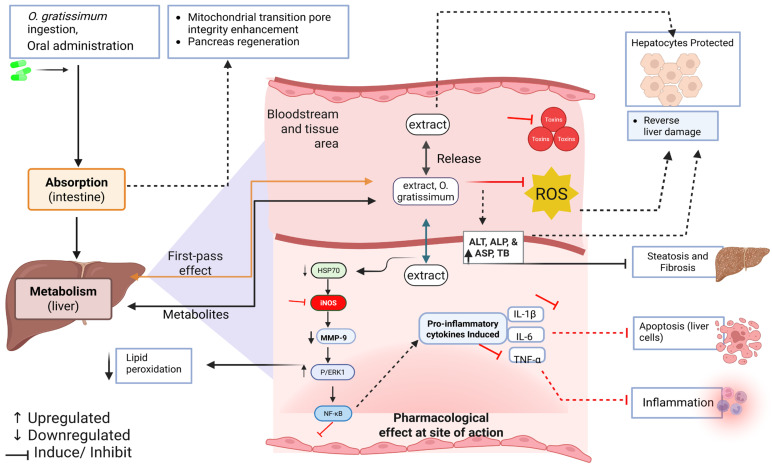
Mechanisms of action for hepatoprotective effects of *O. gratissimum*. Abbreviations: transaminase (AST); alanine transaminase (ALT); alkaline phosphatase (ALP); total bilirubin (TB); reactive oxidative stress (ROS); IL-1β—interleukin 1-β; IL-6—interleukin-6; TNF-α—tumour necrosis factor-α; 70-kilodalton heat shock protein (HSP70); Inducible Nitric Oxide Synthase (iNOS); matrix metalloproteinase-9 (MMP-9); phosphorylated ERK (p/ERK1); nuclear factor kappa-light-chain-enhancer of activated B cells (NF-κB). Figure created using BioRender.com.

Similarly, a study conducted in 2014 by [161] discovered that *O. gratissimum* hepatoprotective effects are dose-dependent; they treated male Wister rats induced with CCl_4._ The study highlights that the *O. gratissimum* extract-treated rats demonstrated liver damage restoration, decreased stenosis and fibrosis, all while increasing hepatises markers such as ALT, ASP, ALP and TB and inhibiting lipid peroxidation [161]. Subsequently, a study conducted by [119], which administered *O. gratissimum* extract to rats induced with hepatocarcinoma cells, show that the administered rats exerted hepatoprotective potential by increasing p/ERK 1 levels, which in turn triggered a survival signal for hepatocytes; see Figure 9 [32,119]. Meanwhile in a different study, root extracts of *Ocimum* plants were found to exert hepatoprotective effects by inducing lipid peroxidation, liver transaminase and malonaldehyde [42]. Moreover, ref. [162] add that *Ocimum* species can induce pro-inflammatory cytokines such as IL-1β, IL-6, TNF-α, and modulate oxidative stress. Figure 18 provides an overview of the molecular mechanisms by which *O. gratissimum* protects the liver.

## 7. Conclusions and Future Studies

*O. gratissimum* is a medicinal plant that has been traditionally used to address a variety of health issues. It is known for its effectiveness against bacterial and fungal infections, as well as its ability to alleviate inflammatory conditions such as pain and swelling. Additionally, it has been used in treating gastrointestinal problems such as indigestion, gastroenteritis, and diarrhoea. In other uses, this plant has also been utilised to manage metabolic disorders like diabetes. Moreover, the plant has other traditional applications in treating psychological conditions, including depression and anxiety. The *O. gratissimum* plant is also valued for its organoleptic properties, such as enhancing the flavours of various African dishes, particularly in West African nations like Nigeria, and creating edible meals to fight malnutrition in African countries.

The variety of bioactive compounds present in *O. gratissimum* enables the plant to have many therapeutic activities. Different kinds of phytochemical mass spectrometry profiling, such as gas chromatography and liquid chromatography, have helped researchers quantify and discover a variety of bioactive compounds. Among these, *O. gratissimum* is known to be a source of essential oils and vitamins, most of which are extracted from its leaves. Furthermore, alkaloids, phenols, fatty acids and alcohols are some of the important bioactive compounds contributing towards the therapeutic effects of *O. gratissimum.* Additionally, *O. gratissimum* is associated with a variety of essential oil clusters such as hydrocarbonated monoterpenes, oxygenated monoterpenes, hydrocarbonated sesquiterpenes and oxygenated sesquiterpenes. Moreover, essential oils make up the plant’s aromatic distinctive smell. Thymol, a volatile essential oil, is one of the bioactive constituents which is responsible for a variety of therapeutic activities. Subsequently, additional nonvolatile bioactive compound constituents include rosmaric acid, apigenin and luteolin, which are essential in contributing towards anti-inflammatory and antimicrobial potential.

*O. gratissimum* therapeutic potential activities have been discovered through different kinds of studies conducted in vivo or in vitro, which have demonstrated the potential effects bioactive compounds have on regulating biological, pharmacological and molecular mechanisms. Studies have shown that *O. gratissimum* has antioxidant potential, by scavenging free radicals such as DPPH, NO, and H_2_O_2_. Moreover, thymol constituent was discovered to be a key player in scavenging potential and in anti-inflammatory potential. The pharmacological and biological molecular mechanisms of *O. gratissimum* are primarily driven by its anti-inflammatory and antioxidant properties. The impact of these pharmacological properties ranges from modulating oxidative stress to activating p/ERK 1 levels, PI3K/Akt, and NRF2 signal pathways.

As a medicinal plant, *Ocimum gratissimum* is highly valued worldwide, particularly in Africa. The plant contains different bioactive compounds and vitamins which are essential in maintaining the body’s homeostasis. *O. gratissimum*, *a* medicinal plant, is a good source of vitamins and therapeutic benefits; further clinical studies are needed. Clinical studies can incorporate the effectiveness of various bioactive compounds of *O. gratissimum* to further assess their benefits in various therapeutic applications. Moreover, further studies on the molecular mechanisms of *O. gratissimum* are needed to elucidate the effects of its bioactive compounds at the molecular level.

## Figures and Tables

**Table 2:** Pharmacological activities of *O. gratissimum* from recent studies.

No.	Pharmacological Activity	Plant Part Used	Extract Compound	Dosage (Experimental Usage)	Type of Study	Methods and Biological Models (Assays)	Findings/Outcomes	References
1.	Antibacterial	Leaves	Aqueous, n-Hexane and ethanol	100 mg/mL	In vivo	*Escherichia coli*, *Salmonella typhi*, *Proteus vulgaris*, *Shigella flexneri*, *Citrobacter freundi*, *Morganella morganii* bacteria were subjected to disc diffusion and agar well diffusion	The ethanol of *O. gratissimum* exhibited strong inhibition properties against *E.coli.*	[85]
2.	Antibacterial: Microbial	Leaves	Aqueous and 70% ethanol	100 mg/mL of extract	In vitro	(*Vibrio* cholerae strains (C6123, E7919, and R1995); Deoxycholate agar and nutrient agar	Extracts exhibited antibacterial activities against *Vibrio* strains through the augmentation of Vitamin C of *O. gratissimum*. Moreover, the ethanolic extract inhibited the *Vibrio* species better compared to the aqueous extract.	[96]
3.	Antibacterial: Fungal	Leaves and branches	70% ethanol	512 µg mL^−1^ of extract	In vivo	*Escherichia coli*, *Staphylococcus aureus*, *Klebsiella pneumoniae*, *Acinetobacter baumannii*, *Pseudomonas aeruginosa*, *Enterococcus faecalis*, and *Proteus mirabilis* were subjected to agar well diffusion and Disc diffusion	Fungus, *Nigrospora oryzae* UILRZ1, extracted from the DNA fragment ITS1 and ITS4 primer pairs, of *O. gratissimum* leaves and branches, showed increased antibacterial activity against the chosen bacterial species, particularly affecting *Staphylococcus aureus*.	[49]
4.	Antidiabetic	Leaves	Aqueous	0–10 mg/mL	In vitro	FRAP, DPPH, and iron chelating	*O. gratissimum* extracts exhibited higher antioxidants output, FRAP, DPPH, indicating potential to reduce and modulate oxidation agents.	[22]
5.	Anti-inflammation	Leaves	Nitric acid and hydrochloric acid	2 g of the extract sample	In vivo	Male Wistar rats treated with inclusion of *O. gratissimum* extracts, conducted an ELISA assay	Wistar rats administered with *O. gratissimum* extract observed a significant increase in serum immunoglobulins G & M, showing improved signs of anti-inflammatory activities.	[29]
6.	Antioxidants	Leaves	Methanol	200, 400 and 600 mg/kg of extract	In vivo	Male Wistar rats, induced with benign prostatic hyperplasia and administered with subcutaneous testosterone propionate	Extracts that the Wistar rats were treated with demonstrated effects in increasing catalase activity and declining malondialdehyde levels, demonstrating *O. gratissimum* extracts’ ability to reduce oxidative stress and improve antioxidant defence mechanisms.	[108]
7.	Antioxidants	Leaves and roots	Aqueous	200 µL, 40 µL and 160 µL	In vitro	*O. gratissimum* supplemented to growth nutrition under photosynthetic photon flux density at 150 µmol m^−2^ s^−1^	*O. gratissimum* exposure to the UV-A radiation enhances antioxidant activities when measured under DPPH, FRAP and oxygen radical absorption capacity.	[113]
8.	Antioxidants	Leaves	Aqueous	3–21 w/v%	In vitro	DPPH, nitric oxide, reducing power, and total antioxidant capacity	Hot aqueous extract has an impact in increasing the antioxidant properties of *O. gratissimum* and reducing resistance (*p* ˂ 0.05).	[79]
9.	Antioxidants	Leaves	Aqueous	0–2.5 mg/mL	In vitro	Carbohydrate-hydrolysing enzymes inhibitory assays	*O. gratissimum* extracts demonstrated higher inhibition of α-amylase (IC_50_: 0.47 mg/mL), and slight inhibition to the α-glucosidase (IC_50_: 9.09 µg/mL).	[22]
10.	Anti-repellent	Whole plant	essential oils	1 µL analysed in GC-MS	In vivo	*Callosobruchus chinensis* subjected to the fumigant bioassay method	Exposure to the *O. gratissimum* extract demonstrated 100% toxicity and death rate of *Callosobruchus chinensis* and healthy growth of grains, signifying that *O. gratissimum* has anti-insectidal properties.	[127]
11.	Anti-repellent	Stem	Ethanol, acetone and aqueous	2 g of extract	In vivo	Mosquito Larvae subjected to larvicidal bioassay and mosquitocidal bioassay	*O. gratissimum* ethanol extract repelled 37.5% of mosquitoes and demonstrated 80% larvicidal effects on mosquitoes.	[30]
12.	Anti-repellent: Insecticidal	Whole plant	-	-	In vitro	*Apolygus lucorum* were subjected to a choice assay	Study results demonstrate that *O. gratissimum* tea plantation reduced the abundance of insects *A. lucorum*, and that natural smell of plant flowers acted as repellents to the insects.	[58]
13.	Hypolipidemic	Leaves	Methanol	200, 400 and 600 mg/kg of extract	In vivo	Male Wistar rats, induced with benign prostatic hyperplasia: Evaluated for total cholesterol using total oxidase, glycerol phosphate oxidase, and low-density lipoprotein cholesterol	*O. gratissimum*-treated specimen lipid profile reduced triglycerides, lowered levels of density lipoprotein cholesterol and increased levels of high-density lipoprotein cholesterol; these findings demonstrate extract-ability for modulate lipid profiles.	[108]
14.	Nootropic/Cognitive enhancement properties	Leaves	Ethanol	150–300 mg/kg, p.o	In vivo	Male Wister rats occluded the middle cerebral artery and underwent reperfusion	Study revealed that *O. gratissimum* ethanol extract has neuroprotective effects by increasing cerebral infarction volume and lipid peroxidation, and decreasing glutathione peroxidase and superoxide dismutase in the brain.	[129]
15.	Anti-convulsant	Leaves	Ethanol	300 mg/kg	In vivo	Albino rats,	Albino rats treated with *O. gratissimum* extract showed and were observed to have isoniazid-induced convulsions compared to the control group of Albino rats, which were treated with picrooxin.	[130]
16.	Organoleptic	Leaves	Hexane	-	In vitro	Hydro-distillation method	The sensory evaluation of the study discovered that *O. gratissimum* concoction spice was significantly preferred due to taste, texture, colour, and flavour, with the acceptability proportion significantly differing from products such as mayonnaise and salad cream.	[47]
17.	Organoleptic	Leaves	-	-	In vitro	Food physio-biochemical analysis	The study found that the acceptability of the *O. gratissimum* extract protein biscuit had the highest level of sensory satisfaction, with 60–70% of participants replacing wheat flour biscuit with *O. gratissimum*	[68]
18.	Organoleptic	Leaves	-	-	In vitro	Hedonic test	The study discovered that 50% of participants preferred the *O. gratissimum* flavoured recipes and sauces compared to other ingredients	[131]
19.	Toxicity/Corrosion	Leaves	Ethanol	300 mL of 15 g extract	In vitro	Water-in-diesel emulsions, a mild steel engine, and conducting gravimetric and surface probe experiments	*O. gratissimum* extract demonstrated a 91.5% effectiveness in preventing corrosion of mild steel when used in a water–diesel emulsion.	[132]

## Data Availability

No new data were created or analyzed in this study. Data sharing is not applicable to this article.
